# Analysis of 36,361 whole genome sequenced samples from the Alzheimer's Disease Sequencing Project (ADSP)

**DOI:** 10.1002/alz70855_106038

**Published:** 2025-12-24

**Authors:** Bilcag Akgun, Seung Hoan Choi, John J. Farrell, Dongyu Wang, Nicholas A. Kushch, Jiemin Yang, Kyle M Scott, Congcong Zhu, Susan H. Slifer, Kara L Hamilton‐Nelson, William S Bush, Adam C. Naj, Jonathan L Haines, Gina M. Peloso, Eden R. Martin, Brian W Kunkle, Anita L. DeStefano, the Alzheimer's Disease Sequencing Project (ADSP)

**Affiliations:** ^1^ John P. Hussman Institute for Human Genomics, University of Miami Miller School of Medicine, Miami, FL, USA; ^2^ Department of Biostatistics, Boston University School of Public Health, Boston, MA, USA; ^3^ Biomedical Genetics, Department of Medicine, Boston University Medical School, Boston, MA, USA; ^4^ University of Miami Miller School of Medicine, Miami, FL, USA; ^5^ Department of Population and Quantitative Health Sciences, School of Medicine, Case Western Reserve University, Cleveland, OH, USA; ^6^ Department of Biostatistics, Epidemiology, and Informatics, and the Penn Neurodegeneration Genomics Center, Department of Pathology and Laboratory Medicine, University of Pennsylvania Perelman School of Medicine, Philadelphia, PA, USA; ^7^ Department of Population and Quantitative Health Sciences, Institute for Computational Biology, Case Western Reserve University, Cleveland, OH, USA

## Abstract

**Background:**

The Alzheimer's Disease Sequencing Project (ADSP) aims to uncover genomic variants that increase risk for or protect against Alzheimer's disease (AD) across various ancestral populations. The most recent ADSP whole genome sequencing (WGS) data release (R4) includes data from 36,000+ individuals across 37 study cohorts.

**Method:**

Extensive quality control was conducted at the genotype, variant, and sample levels. Analyses focused on AD and related dementias (ADRDs) with cases and controls restricted to age ≥55 years. Two main genetic association approaches were applied: 1) *Population‐specific analysis*‐ Genetic similarity clusters for defining populations were identified using a Gaussian mixture model. Analyses included generalized linear mixed model single‐variant tests for common variants with SAIGE; meta‐analysis of common variants across populations using METASOFT; and set‐based rare variant analyses for coding and noncoding regions with STAAR, followed by meta‐analysis using MetaSTAAR. 2) *Pooled population analysis*‐ Gene‐based association tests including common and rare variants were conducted using GENESIS/R, accounting for sex, technical covariates, population structure, and empirical relationships. Rare variant analyses aggregated predicted loss‐of‐function and deleterious missense variants, with Bonferroni correction.

**Result:**

For the population‐specific analysis, we defined nine clusters of individuals (8,697 ADRD cases and 14,758 controls) based on genetic similarity using high‐quality variants. The meta‐analysis of common variants identified two known ADRD‐associated loci (*APOE* and *CR1*) and four novel genomic loci (*LMO1, FHIT, RPS3AP52*, and *AC013762.1*) that reached genome‐wide significance (*p* <5×10^‐8^). For the pooled analysis, common variant associations confirmed the signals in *APOE* and *CR1*. Additionally, rare variant gene‐based analysis identified *KIRREL1* (*p* = 1.01×10^‐6^) and *TNFRSF10B* (*p* = 2.25×10^‐6^) as significantly associated in population‐specific meta‐analysis and *TNS1* as suggestively associated with ADRD (*p* = 3.05×10^‐6^) in pooled analysis.

**Conclusion:**

Analyses of ADSP WGS data identified a novel genome‐wide significant association at *LMO1* (11p15), a gene involved in transcriptional regulation with potential relevance to neuronal health, and a suggestive association at *TNS1* (2q35), previously linked to cognitive decline in non‐demented older adults. Further studies in diverse populations from the ADSP are expected to uncover additional common and rare variant associations, offering new insights into ADRD pathogenesis.

